# An Experimental Evolution Test of the Relationship between Melanism and Desiccation Survival in Insects

**DOI:** 10.1371/journal.pone.0163414

**Published:** 2016-09-22

**Authors:** Subhash Rajpurohit, Lisa Marie Peterson, Andrew J. Orr, Anthony J. Marlon, Allen G. Gibbs

**Affiliations:** School of Life Sciences, University of Nevada, Las Vegas, NV 89154, United States of America; National Cancer Institute, UNITED STATES

## Abstract

We used experimental evolution to test the ‘*melanism-desiccation*’ hypothesis, which proposes that dark cuticle in several *Drosophila* species is an adaptation for increased desiccation tolerance. We selected for dark and light body pigmentation in replicated populations of *D*. *melanogaster* and assayed several traits related to water balance. We also scored pigmentation and desiccation tolerance in populations selected for desiccation survival. Populations in both selection regimes showed large differences in the traits directly under selection. However, after over 40 generations of pigmentation selection, dark-selected populations were not more desiccation-tolerant than light-selected and control populations, nor did we find significant changes in mass or carbohydrate amounts that could affect desiccation resistance. Body pigmentation of desiccation-selected populations did not differ from control populations after over 140 generations of selection, although selected populations lost water less rapidly. Our results do not support an important role for melanization in *Drosophila* water balance.

## Introduction

Pigmentation in insects is extremely diverse, both among and within species [1,2,3,4,5]. In addition to differences in color, insects can differ in the deposition of melanin, a dark polymer of dopa derivatives. Several adaptive hypotheses have been proposed for variation in melanization [3,6,7]. These include behavioral benefits (crypsis, sexual selection, etc.) and physiological benefits, including thermoregulatory capacity and resistance to abrasion, ultraviolet radiation, infection and desiccation [8,9,10,11]. In recent years, several research groups have used *Drosophila* to investigate the functional significance of melanization, particularly in the context of desiccation stress and water balance. Water balance is a general physiological problem for insects, because their large surface area:volume ratio makes insects susceptible to water loss through the cuticle.

The genus *Drosophila* provides an excellent system in which to examine the function of melanization. Species differ widely in their body pigmentation, and pigmentation mutants have been identified in several species. For example, *ebony* mutants of *D*. *melanogaster* are more resistant to desiccation stress than wildtype flies, whereas *yellow* mutants of multiple species are less desiccation resistant [12]. Within species, darker populations tend to be more desiccation resistant [13,14,15], and, within populations, darker pigmentation is also associated with increased desiccation resistance [16].

A series of recent studies have examined parallel clines in desiccation tolerance and body melanization in several *Drosophila* species from the Indian subcontinent [17]. Populations of *D*. *melanogaster* from higher (and dryer) latitudes and altitudes on the Indian subcontinent are darker and more resistant to desiccation [9,15,17,18,], suggesting that differences in pigmentation are correlated. This idea is supported by the findings that darker phenotypes of *D*. *melanogaster* and other species lose water less rapidly than lighter phenotypes [19,20]. A potential mechanistic explanation for these correlations is the hydrophobic nature of melanin. Like epicuticular hydrocarbons, melanin may decrease the permeability of the cuticle to water [21], or melanin may thicken the cuticle and increase the distance for diffusion of water through the cuticle.

By contrast, natural populations of *D*. *americana* from a longitudinal cline in North America are darker in more humid areas [22], suggesting that selection promoting the pigmentation cline in *D*. *americana* might be different from that in other *Drosophila* species. More recently, Matute and Harris [23] reported that populations of *D*. *yakuba* from the coast of the Gulf of Guinea showed significant differences in pigmentation but not in desiccation tolerance. Thus, investigations of the potential link between melanization and water balance in natural populations have reached conflicting conclusions. In addition, other traits related to water balance, such as cuticular hydrocarbons, may also exhibit clines [24].

A central problem for studies of clinal variation is that environmental factors often co-vary, making it difficult to distinguish which factor (or combination thereof) is responsible for the cline. For example, a recent synthesis of clinal variation in Indian populations of *Drosophila* [17] found that desiccation resistance was most highly associated with the coefficient of variation in monthly temperature, which was highly correlated with mean annual temperature, mean relative humidity, and the coefficient of variation of monthly relative humidity. Thus, parallel clines in traits may arise from independent selection exerted by parallel clines in environmental variables. For example, selection for reduced water loss in dry environments may coincide with selection for thermoregulatory capability, resulting in co-evolved differences in these traits.

Experimental evolution provides a means to manipulate environmental factors independently and to rigorously test whether correlated variation observed in natural populations has a physiological or genetic basis. Direct comparison of laboratory and natural systems provides the opportunity to identify and test hypotheses regarding natural selection in the field [25,26,27,28,29,30]. If laboratory and comparative studies provide similar results, this is corroborative evidence that selection is acting as we thought in nature [31]. When different results are obtained, then something may be missing in our understanding of one or both environments [26]. A recent pigmentation selection experiment using *D*. *melanogaster* supported the melanism-desiccation hypothesis [20]. Populations selected for darker pigmentation were more desiccation tolerant than controls. However, potential differences in melanism of desiccation-selected *Drosophila* have not been investigated.

In this study, we used experimental evolution to test the hypothesis that melanism and desiccation tolerance are functionally associated in *D*. *melanogaster*. Previous studies have demonstrated that natural populations of *D*. *melanogaster* harbor significant genetic variation for both traits. Pigmentation and desiccation tolerance each respond rapidly to selection in the laboratory (pigmentation: [20,32]; desiccation tolerance: [33,34]). We reasoned that selecting populations for darker or lighter pigmentation should result in populations with greater or lesser desiccation tolerance, respectively. Conversely, selection for increased desiccation tolerance should result in darker populations of *Drosophila*. Our results contradict these predictions. Desiccation-selected flies were not darker than controls, and pigmentation-selected populations exhibited relatively small differences in desiccation resistance that were not consistent with our predictions. We also examined potential correlated responses to selection on other traits associated with water balance, such as body size and carbohydrate content, to determine whether these may have affected our results.

## Materials and Methods

### Fly collection and maintenance

No special permission is required to collect the fruit fly *Drosophila melanogaster* from fruit orchards in the United States. The collectors obtained verbal permission to trap flies from the owners of the orchards. This species is not endangered. Pigmentation-selected lines were founded from ~400 females collected in Gilcrease Orchard, Las Vegas, Nevada, USA (36.30° N; 115.24° W) in 2008, and desiccation-selected lines were founded from a population (~400 individuals) collected in Terhune Orchard, New Jersey, USA (Lat 40.33° N; Long 74.72° W) in 1999.

### Selection for body melanization and desiccation tolerance

The selection protocols for desiccation and pigmentation selection were detailed in Gefen et al. [35] and Rajpurohit and Gibbs [32], respectively. Selecting for pigmentation entailed artificial selection in the laboratory, in which the darkest or lightest 10% females, as chosen by the primary author, were allowed to reproduce each generation. Flies were collected within one day of eclosion and kept 1 week in mixed sex vials, for aging and mating. 200 one-week-old females were randomly selected from each population, and the 20 darkest or lightest (based on the method developed by David et al. [36]) were allowed to lay eggs for the next generation. Three replicate dark-selected (D_PIG_) and light-selected (L_PIG_) populations were created from the initial founding population, along with three control (C_PIG_) populations, for which 20 breeding females were selected randomly from the population each generation. Pigmentation and tergite area data were collected after 40 generations of selection; other data for these populations were collected after 52 generations of selection.

To select for desiccation resistance, three replicated populations (D) were selected for desiccation tolerance, and three control populations were maintained without desiccation stress (F, continuous access to food and water). For desiccation selection, ~10,000 flies were exposed to low humidity conditions (no food, in the presence of desiccant) each generation, and the ~10% individuals that survived the longest were allowed to recover and produce offspring. After 30 generations of increasingly long selection bouts (~15 hr in the first selection generation, and >35 hr in the 30^th^ generation), the D populations were subjected to a 24 hr “maintenance” desiccation period each generation (~20% mortality). Periodic desiccation assays have revealed that nearly all fed control flies die within 24 hr, whereas the D populations have maintained their desiccation resistant phenotype. These populations had undergone 135–145 generations of selection when the experiments were performed.

### Egg collection for experimental assays

Adults from each population were transferred to empty 175-ml bottles for one hour. The bottles were covered with a 35x10 mm Petri dish containing grape agar as a substrate for egg laying. Sets of 60 eggs were collected in replicates and placed in food vials containing approximately 10 ml of cornmeal-yeast-sucrose media. To avoid potential parental effects, the populations were kept off selection for one generation before performing any analyses. Newly eclosed flies were collected and aged on fresh media for 4 days before subjecting them to assays.

### Desiccation resistance

Four to five-day old virgin flies were briefly anesthetized with CO_2_, transferred to empty vials in groups of five, and restricted to the lower half of the vials by a foam stopper. Silica gel was then added above the stopper to maintain low humidity, and the vial was sealed with Parafilm. Mortality was recorded at hourly intervals until all flies were dead. Both sexes were assayed in the pigmentation-selected populations, but only females for the desiccation-selected populations.

### Tergite pigmentation scoring and area measurements

For the measurements of abdominal tergite pigmentation and size, we used whole mount abdomens prepared on transparent glass slides. The mounted abdomens were imaged using a Nikon digital camera attached to a dissecting microscope. We collected data for abdominal pigmentation and total dorsal abdominal area based on an existing method with slight modifications [37]. We scored gray score for all 5 abdominal tergites together (T2-T6). Briefly, the upper thoracic and abdominal cuticle were dissected away from the body and flattened on a microscope slide under a slide cover. A calibrated scale image was taken before the sample images (without changing magnification between the scale image and sample images). The images were then analyzed using ImageJ software (http://rsbweb.nih.gov/ij/). These measurements used seven-to-ten day old flies of both sexes. Pigmentation-selected populations were assayed after 40 generations of selection, while desiccation-selected populations were assayed after ~140 generations.

### Wet mass, dry mass and water content

Individual four-day old flies were weighed on a Cahn C-30 microbalance. To estimate wet weight, the flies were frozen at -20°C and weighed immediately after removal from the freezer. All samples were measured within one week to avoid freezing-related dehydration. Dry weights were measured as the weight after drying at 50°C overnight. Total body water content was estimated as the difference between masses before and after drying at 50°C. For pigmentation selection lines (L_PIG_, C_PIG_ and D_PIG_) we studied both sexes. For desiccation selection lines (D and F) we had just male samples.

### Carbohydrate assays

Previous desiccation selection studies in *D*. *melanogaster* suggest a correlation between glycogen storage and desiccation tolerance [35,38,39]. For carbohydrate measurements, flies were frozen at –20°C. After thawing, the flies were sexed, homogenized in 200 μl 0.05% Tween-20, and incubated at 70°C for 5·min. The samples were then centrifuged for 1·min at 16·000·***g***, and the supernatants removed and frozen. Carbohydrate content (trehalose and glycogen) was measured following the methods used by [35]. These measurements were done only for L_PIG_, C_PIG_ and D_PIG_ populations.

### Respirometry

Water-loss rates and metabolic rates were measured using flow-through respirometry (TR-2 respirometer; Sable Systems, Las Vegas, Nevada, USA). Groups of 10–20 flies were placed in 5ml glass/aluminum chambers, and dry CO_2_-free air was pumped through the chambers at a flow rate of 50 ml min^-1^ to an LI-6262 infrared CO_2_ sensor (Li-Cor Biosciences, Lincoln, Nebraska, USA). Recordings began approximately 90 minutes after placement in the respirometer. Metabolic and water-loss rates were calculated from CO_2_ and water vapor released by flies into the air stream. The humidity sensor was calibrated by injection of small drops of water (0.5–3.0 nl) into the air stream, and the CO_2_ detector was calibrated according to the manufacturer’s instructions using 100 ppm span gas. Datacan V software (Sable Systems, NV, USA) was used for data collection and analysis. Respirometry measurements were performed on both sexes of pigmentation (L_PIG_, C_PIG_ and D_PIG_) as well as desiccation selection lines (D and F).

### Statistical analyses

We used Statistica v7.1 to analyze our data. Desiccation- and pigmentation-selected populations (and their respective controls) were analyzed separately. We used mixed-model analyses of variance (ANOVA), with selection and sex as fixed main effects and replicate population as a random variable nested within selection treatment. When interaction terms were not statistically significant, we re-ran ANOVAs without these interactions. The conclusions reached did not change, so we have presented statistical analyses with all interactions included in the models. Within selection treatments, replicate populations differed significantly for several traits. For consistency, figures therefore show data for each replicate population. Desiccation-resistance data for the D and F populations were analyzed using log-ranks tests, with censoring for missing data points.

## Results

### Pigmentation

**Fig 1:**
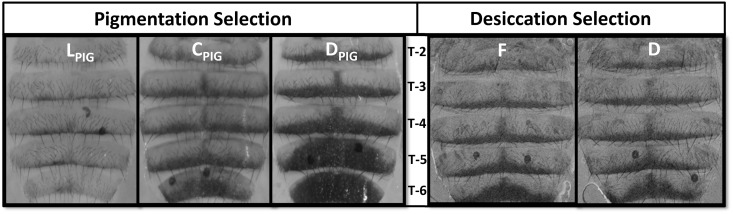
Representative images of abdominal tergites (T2-T6; anterior to posterior) in pigmentation-selected populations after 40 generations of artificial selection (left panels) and >140 generations of laboratory natural selection for desiccation tolerance (right panels). Only female images are shown here.

**Fig 2:**
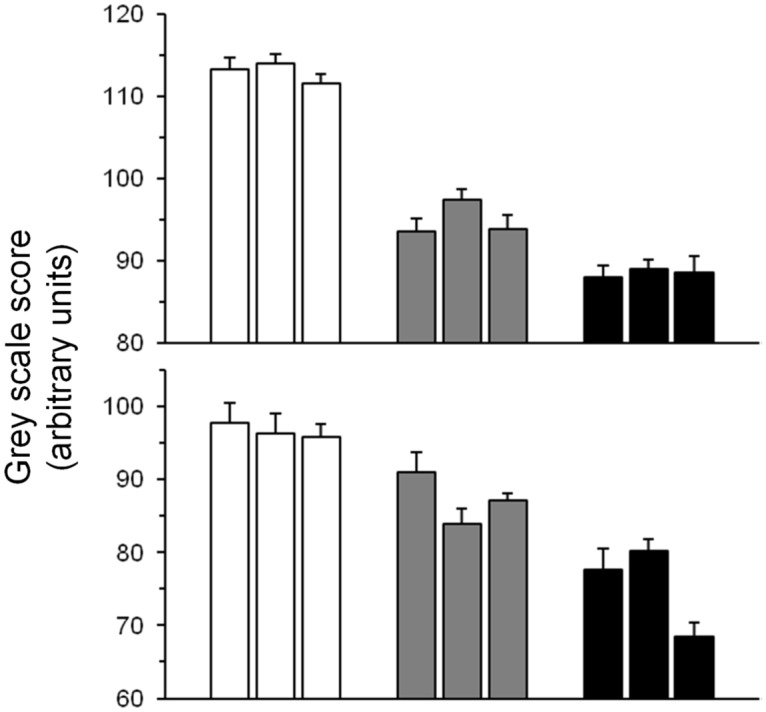
Pigmentation status (as gray scale score) in pigmentation-selected populations after 40 generations of artificial selection. Higher gray scores indicate lighter pigmentation. Upper panel, females; lower panel, males. Open bars, L_PIG_; gray bars, C_PIG_; black bars, D_PIG_. Data are means (±SE). For both sexes, Tukey post-hoc tests revealed significant differences for all pairwise comparisons (P<0.0005): L_PIG_ > C_PIG_ > D_PIG_. For each sex, n = 9–10 per replicate population.

**Fig 3:**
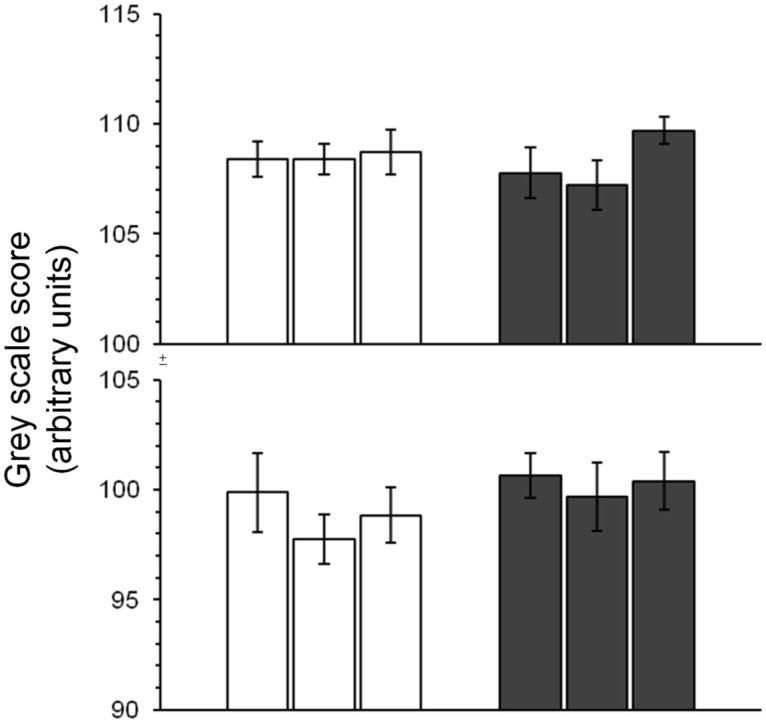
Pigmentation status (as gray scale score) in D and F populations after ~140 generations of laboratory natural selection. Upper panel, females; lower panel, males. Open bars, fed (F) controls; filled bars, desiccation-selected (D) populations. Data are means (±s.e.). For each sex, n = 15–20 per replicate population.

Body tergite melanization for females from pigmentation- and desiccation-selected populations are shown in Fig 1. Gray scale scores of populations selected for abdominal pigmentation (D_PIG_, C_PIG_ and L_PIG_) showed a significant response to selection (Fig 2; F_2,161_ = 99.91; P<0.00003; see S1 Table, available online). Although females were the direct target of selection in the pigmentation-selection regime, males also evolved differences in pigmentation (S1 Table). Besides selection and sex as significant main effects, selection*sex interaction effects were also significant (F_6,161_ = 3.24; P<0.005; S1 Table, available online). In contrast, no differences in abdominal tergite pigmentation were detected between desiccation-selected and control populations after ~135 generations of selection (Fig 1, right panels; Fig 3; S2 Table, available online; F_1,198_ = 1; P<0.37).

### Desiccation resistance

**Fig 4:**
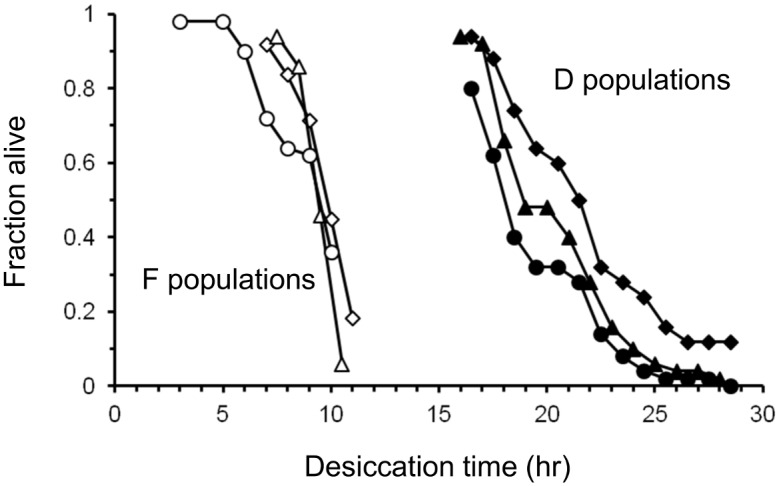
Desiccation survival in D and F males after ~135 generations of laboratory natural selection. Open symbols, F males; filled symbols, D males. Each symbol represents a different replicate population. n = 48–50 per replicate population.

In assays of desiccation-selected (D) populations, most fed control (F) flies had died within 11 hours under desiccating conditions, whereas in D flies little mortality occurred before 15 hours (Fig 4). Some survival data were missing at the tails of the F and D survival curves, so we compared each replicate D and F population to all of the other populations using log-ranks tests, with a sequential Bonferroni correction for multiple comparisons. All D replicates survived longer than all F replicates. Every pair-wise comparison of a D and an F population was statistically significant, while no comparisons between replicates within a selection treatment were significant (S3 Table). When replicate populations were pooled, desiccation resistance of D flies was significantly greater than that of F flies (log-ranks test; P < 10^−5^).

**Fig 5:**
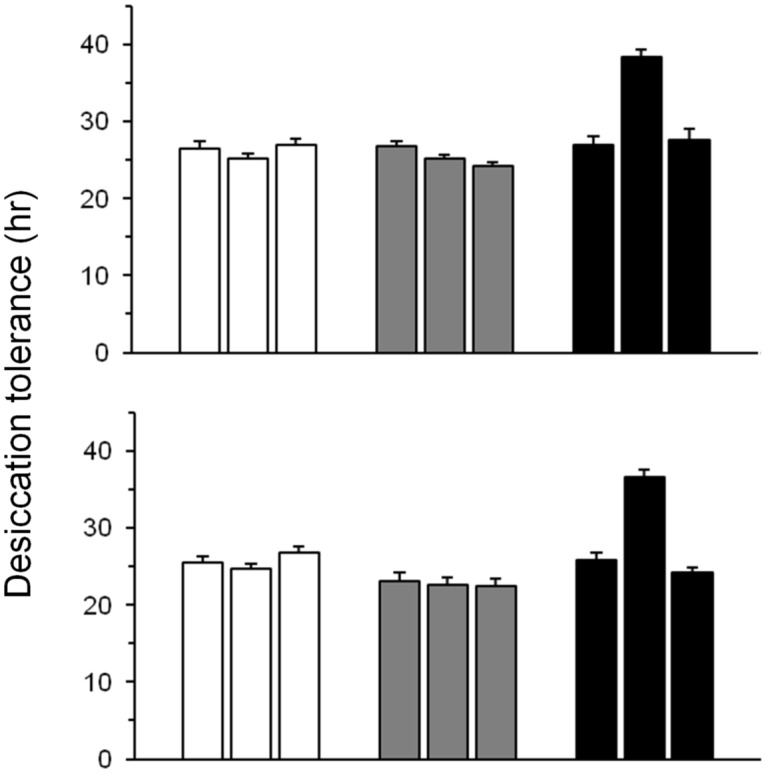
Survival under desiccating conditions in pigmentation-selected populations after 52 generations of artificial selection. Upper panel, females; lower panel, males. Open bars, L_PIG_; gray bars, C_PIG_; black bars, D_PIG_. Data are means (±SE). For each sex, n = 10–15 per replicate population.

No data were missing from the desiccation survival assays of pigmentation-selected populations, so we were able to calculate mean survival times. Pigmentation selection did not affect desiccation resistance (Table 1; F_2,200_ = 1.81, P>0.24), although replicates (nested within selection) did differ among each other. Closer inspection of the data revealed that one D_PIG_ replicate population survived nearly 50% longer than the other 8 populations (Fig 5). As expected, significant differences in desiccation tolerance between the sexes were found in pigmentation-selected populations, with males dying earlier than females.

**Table 1:** Nested ANOVA results for desiccation tolerance in pigmentation-selected populations and controls. For each sex, n = 10–15 flies per replicate population.

Parameters	SS	d.f.	MS	F	*p*
Selection	1277.4	2	638.7	1.81	0.24
Replicate(Selection)	2112.5	6	352.1	78.0	0.00002
Sex	172.7	1	172.7	38.2	0.0008
Replicate(Selection*Sex)	27.1	6	4.5	0.499	0.81
Selection*Sex	41	2	20.5	4.53	0.063
Error	1809	200	9		

### Wet and dry mass, carbohydrate and total water content, tergite area

Previous work in our D populations [35,39] and other desiccation-selection studies [38,40] have implicated increased glycogen storage as contributing to desiccation tolerance. Glycogen storage itself will increase overall body mass, and larger flies will generally survive desiccation longer. We therefore examined several measures of size (mass, tergite area) to determine whether either pigmentation or desiccation selection resulted in correlated changes in size.

We analyzed wet mass, dry mass and total water content in pigmentation-selected populations. For each of these size measures, females were ~30% larger than males. ANOVAs revealed no differences in wet mass of D_PIG_, C_PIG_ and L_PIG_ populations (selection F_2,520_ = 1.759; P<0.25; S4 Table), or water content (selection F_2,520_ = 0.95; P<0.5; S4 Table), but a significant difference was observed in dry mass (selection F_2,520_ = 7.18; P<0.03; S4 Table). A Tukey post-hoc test revealed that control C_PIG_ populations had lower dry masses than either of the pigmentation-selected treatments. Dry mass measurements were available for males only from the D and F populations. D males weighed nearly 30% more than F males (0.303 mg ±0.010 s.e. for D males vs. 0.235 mg ±0.005 s.e. for F males). This difference was statistically significant (F_1,54_ = 42.0; P<0.003; n = 10 flies per replicate population).

Pigmentation-selected populations did not differ in carbohydrate content from their controls or from each other (S1 Fig; S5 Table). Females accumulated more carbohydrate than males. A nested ANOVA revealed significant differences in total carbohydrate content among replicate populations when they were nested within selection*sex (S5 Table; F_6,124_ = 3.51; P<0.003). Thus, replicates within a given selection treatment sometimes differed, but there were no overall differences between treatment groups. We did not measure carbohydrate levels in the D populations in this study, but prior experiments [35] and subsequent studies [39] reveal D flies have higher carbohydrate levels than F flies.

Tergite area did not differ among pigmentation-selected populations, although differences approached statistical significance (selection F_2,161_ = 4.24; P<0.08; S6 Table). Further inspection of the data revealed that this pattern was driven by a trend in females: L_PIG_<C_PIG_<D_PIG_ (S2 Fig). This pattern also approached statistical significance when the sexes were analyzed separately (female selection F_2,81_ = 4.83; P<0.06). In desiccation-selected populations, selection and sex significantly affected tergite area (selection F_1,198_ = 26.0; P<0.007; sex F_1,198_ = 1716; P<0.0001; S7 Table, available online). Females had larger tergite areas than males, and D flies were larger than F flies (S3 Fig, available online).

### Metabolic rate and water loss rate

**Fig 6:**
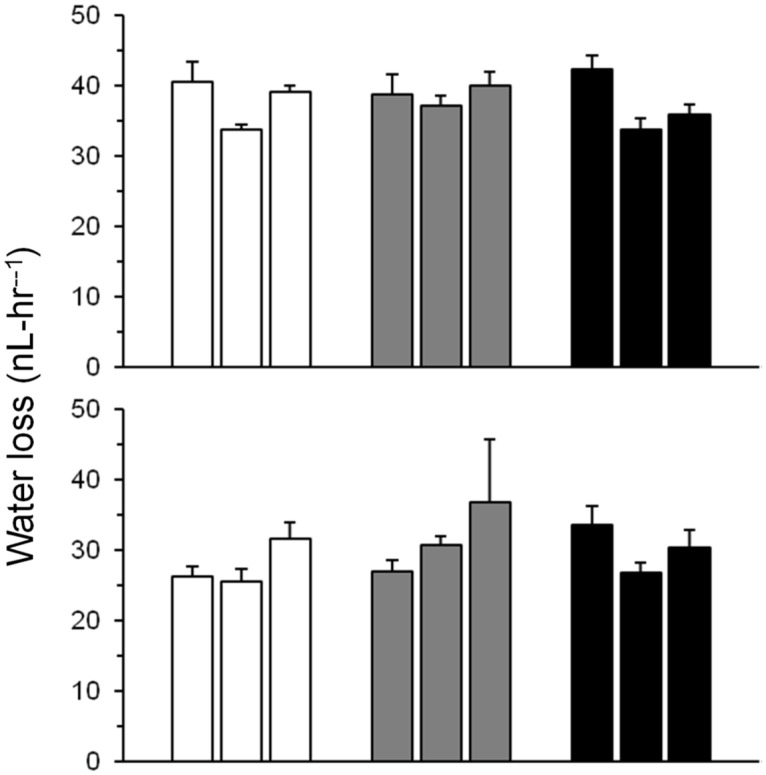
Water-loss rates in pigmentation-selected populations after 52 generations of artificial selection. Each bar represents mean (±SE) for a replicate population. Upper panel, females; lower panel, males. Upper panel, females; lower panel, males. Open bars, L_PIG_; gray bars, C_PIG_; black bars, D_PIG_. Data are means (±SE). For each sex, n = 3–7 per replicate population.

We found no differences in water-loss rate (WLR) and metabolic rate (MR) among pigmentation-selected flies (WLR F_2,90_ = 0.35; P>0.6; MR F_2,90_ = 0.16; P>0.8; Fig 6; Tables 2 and 3), although significant metabolic rate differences appeared among replicates (nested within selection). Surprisingly, desiccation-selected D flies did not lose water more slowly on an individual basis than F controls (S3 Fig, available online; F_1,60_ = 3.94, P>0.1; S8 Table, available online). Closer inspection of the data indicated that this result was associated with relatively greater variation among male replicates than female (S4 Fig, available online). When sexes were analyzed separately, D females had lower water-loss rates than F females (F_1,30_ = 12.5, P<0.025). It should also be noted that water-loss rates were expressed on a per-fly basis. D males were larger, which could result in lower mass-specific water-loss rates than in F males. However, we did not measure mass and water-loss rates in the same flies, so direct comparisons of mass-specific metabolic rates could not be done.

**Table 2:** Nested ANOVA results for water-loss rate of pigmentation-selected populations and controls. For each sex, n = 3–7 groups of flies per replicate population.

Parameters	Effect (F/R)	SS	df	MS	F	p
**Selection**	Fixed	22.79	2	11.39	0.359	0.71
**Replicate(Selection)**	Random	190.50	6	31.75	3.79	0.065
**Sex**	Fixed	439.66	1	439.66	52.4	**0.00035**
Replicate(Selection*Sex)	Random	50.32	6	8.39	0.859	0.53
Selection*Sex	Fixed	11.80	2	5.90	0.703	0.53
**Error**		878.41	90	9.76		

**Table 3:** Nested ANOVA results for metabolic rate of pigmentation-selected populations and controls. For each sex, n = 3–7 groups of flies per replicate population.

Parameters	Effect (F/R)	SS	df	MS	F	p
**Selection**	Fixed	0.471	2	0.235	0.165	0.85
**Replicate(Selection)**	Random	8.594	6	1.432	7.18	**0.015**
**Sex**	Fixed	12.893	1	12.893	64.5	**0.0002**
Replicate(Selection*Sex)	Random	1.198	6	0.200	0.606	0.73
Selection*Sex	Fixed	0.343	2	0.172	0.858	0.47
**Error**		29.670	90	0.330		

## Discussion

Insects lose >70% of their body water through the cuticle, and melanin is an important cuticular constituent. Along latitudinal and altitudinal transects, parallel clines for desiccation and pigmentation in *Drosophila* suggest that these two traits are functionally associated [9,15,17,18]. Melanin’s hydrophobic nature is consistent with a reduction in cuticular permeability, making this an attractive physiological hypothesis. Other physiological hypotheses include thermoregulation [41,42] and resistance to ultraviolet (UV) radiation or abrasion. Most *Drosophila* are too small to maintain a body temperature different from ambient conditions [43,44], so thermoregulation is an unlikely function in this taxon. Arid regions generally have reduced cloud cover, yet Matute and Harris [23] found that lighter *D*. *yakuba* and *D*. *santomea* were more UV tolerant than darker flies. A role for melanism in abrasion resistance has not been tested yet.

Several previous studies have shown that pigmentation and desiccation resistance both respond rapidly to selection in the laboratory [20,32–34]. If melanism and desiccation resistance are indeed mechanistically linked through differences in cuticular permeability, then selection on either trait should result in correlated responses in the other. We performed two complementary selection experiments, and our results do not support the *‘melanism-desiccation’* hypothesis. However, survival under desiccating conditions is a function of multiple physiological characters, and it is possible that other characters have undergone correlated responses to selection in both pigmentation- and desiccation-selected populations.

Our strongest evidence that melanism has little effect on cuticular water loss is provided by the pigmentation-selected populations. Despite clearly visible differences in appearance (Fig 1), light- and dark-selected flies did not differ in overall water-loss rates (Fig 6). An exception was one D_PIG_ population that was significantly more resistant to desiccation than any of the other populations (Fig 5). This population was not darker than the other D_PIG_ populations; in fact, flies of both sexes were slightly, but not significantly, lighter than those from the other replicates.

Cuticular transpiration and respiration are the primary routes for water loss from insects [45,46]. It is possible that pigmentation-selected lines differed in respiratory water loss in a way that counteracted cuticular water loss differences (lighter populations with higher cuticular water loss could have lower respiratory water-loss rates). Metabolic rates, as measured by CO_2_ production, did not differ among pigmentation-selected populations and their controls (Table 3), suggesting that differences in respiratory water loss did not affect overall water-loss rates. In other studies, relatively inactive desiccation-selected flies do not have lower respiratory water-loss rates than controls [47]. We note that our work and previous studies have used CO_2_ production as indirect measure of metabolism; changes in metabolic fuel source could affect these measurements. Previous studies have found glycogen to be the preferred fuel source in desiccated *Drosophila* [48,49].

Several laboratories have selected for desiccation resistance in laboratory populations of *D*. *melanogaster*. Consistently, these populations evolve reduced water-loss rates [38,35,50,34]. Our study is the first to examine melanism in desiccation-selected *Drosophila*. We found no differences in pigmentation between D populations and their F controls, despite large differences in desiccation tolerance and water-loss rates (Fig 4). As was the case for our pigmentation selection experiment, the lack of the expected correlated response to selection provides evidence that melanism does not significantly affect water balance in *D*. *melanogaster*.

An important consideration in insect water balance is body size—larger individuals are predicted to lose water relatively slowly due to surface area:volume considerations. For example, we found that females, which are larger than males, had greater desiccation tolerance than males. Flies reared on poor quality food as larvae are smaller and lighter in color than well-fed controls [51]. This suggests a tradeoff between resource allocation to pigmentation and other organismal requirements. Such a tradeoff could result in flies selected for lighter pigmentation being larger than dark-selected flies, while melanism could cause D_PIG_ flies to be smaller, but relatively desiccation tolerant, for their size. We therefore compared several indicators of size in pigmentation-selected populations. Flies did not differ in overall mass or water content, although control populations had slightly, but significantly, lower dry mass than either light- or dark-selected populations (S4 Table). Pigmentation selection also did not affect abdominal tergite area (S2 Fig). In fact, there was a trend in females for darker females to be larger, rather than smaller, than lighter females. We conclude that correlated responses in body size cannot explain why selection for darker or light pigmentation did not affect desiccation tolerance.

If overall changes in size do not affect desiccation tolerance, changes in body composition might. As noted above, pigmentation controls (C_PIG_) had lower dry mass than both light- and dark-selected flies. The greater dry mass of L_PIG_ and D_PIG_ flies could reflect preferential storage of resources that affect melanization or desiccation tolerance, Desiccation-selected populations of *D*. *melanogaster* contain more carbohydrates than control populations [35,38,39,40,52]. Glycogen contains bound water equivalent to >3 times its dry mass, and this water is released when glycogen is metabolized [38]. *Drosophila* species in general tend to metabolize glycogen under desiccating conditions [48,49], consistent with the release of bound water when it is required. Thus, we may predict that D_PIG_ flies contain more carbohydrate. Instead, L_PIG_ flies tended to contain more carbohydrate than D_PIG_ or control flies (S1 Fig).

Body size is an important factor in the desiccation-selected populations. Males from desiccation-selected (D) populations had higher dry weights than their fed (F) controls, consistent with previous work [35]. Limited data on females were consistent with this pattern. Both sexes also had larger abdominal tergite areas. Thus, D flies were larger than their controls. Despite this, D females lost water less rapidly than controls on an individual basis (i.e. larger D females lost less water than smaller F controls; S4 Fig). D and F males did not differ in water-loss rate per individual fly, but given their 30% larger dry mass, D males certainly lost water less rapidly per unit of mass and surface area. Reduced metabolic rates may have contributed, but previous work found no differences between similar desiccation-selected and control populations in the first few hours of desiccation stress [53]. We conclude that D flies had lower cuticular permeability than F controls, despite the lack of melanization differences.

Increased body size may be responsible for the relatively high desiccation tolerance of one D_PIG_ replicate population (Fig 5). This population had higher wet mass and water content than all other populations, although dry mass of both sexes approximated the average for all of the pigmentation-selected populations. Thus, this population may have evolved increased water storage, similar to the situation for some desiccation-selected populations [38].

Epicuticular hydrocarbons (HC) provide an important barrier to cuticular transpiration in insects [21], and it is possible that selected populations differed in the amount and/or composition of HC [54]. Such differences have been implicated in latitudinal clines in Indian populations of other drosophilids [7], but not *D*. *melanogaster* [24]. However, longer-term (160 generations), more stringent desiccation selection than performed in this study yielded only minor HC differences [38]. Inter-specific and acclimatory studies reveal no consistent relationships between water-loss rates and HC [55,56,57]. Although we cannot exclude HC differences between selected populations and controls in this study, large differences in cuticular water loss can be achieved without substantial HC differences.

Our pigmentation-selected *Drosophila* populations did not differ in desiccation tolerance (except one D_PIG_ replicate line), in contrast to the findings of Ramniwas et al. [20]). One possible explanation is the different sources used to found selected populations. Clear size differences exist between our populations and those studied by Ramniwas et al. [20]; see Table 2 in that study). Our male flies were smaller, and we found much greater sexual dimorphism than Ramniwas et al. did. Another potential explanation for these conflicting results is that our populations came from a local orchard in the Mojave desert. Our pigmentation-selected populations survived ~24 hours in dry air, longer than fed controls for our desiccation-selected populations, as well as controls in other desiccation selection experiments [38,50]. However, control populations described by Ramniwas et al. [20] also survived ~24 hours of desiccation under similar conditions, suggesting that their founding populations, and ours, may have been adapted to relatively xeric conditions in nature.

Another potentially important explanation for these conflicting results is the design of the selection experiments. Our desiccation and pigmentation-selected populations were each founded from ~400 females from single natural populations of *D*. *melanogaster*. Ramniwas et al. [20] started with a pool of 30 mated pairs each from 6 geographically isolated populations along an altitudinal transect. These populations face different climatic conditions in their natural habitats and exhibit positive correlations between altitude and both melanism and desiccation tolerance [16,58]. Before setting up selection lines from their pooled population, Ramniwas et al. [20]) reared stocks from each natural population for 6–7 generations. Differential reproductive success of some genotypes, or linkage disequilibrium between genes responsible for pigmentation and desiccation tolerance, could have resulted in positive correlations being retained during laboratory selection. Even if melanism and desiccation tolerance are not mechanistically associated, linkage disequilibrium as populations interbred under artificial selection for pigmentation could have resulted in correlated responses in water balance.

The founding populations of these independent selection experiments also have very different colonizing paths and history. *Drosophila melanogaster* originated in Africa and colonized North America relatively recently (<300 years; [59,60,61,62]), whereas they entered Europe over 10,000 years ago and Asia much earlier [59,63]. Genetic polymorphism of these populations could have been reduced through adaptation to new environments, or through demographic events taking place during range expansion, including bottlenecks and founder events [59,64]. Thus, our founding populations and those used by Ramniwas et al. [20] would have had different initial allelic variation. Chromosomal inversions have been repeatedly involved in local adaptation in a large number of animals and plants [65–68]. Differences in chromosomal inversions would also contribute to linkage disequilibrium [69]. Thus, the pool of existing genetic diversity when selection experiments began were likely to have differed, potentially affecting their outcomes.

## Conclusions

We performed complementary selection experiments in *Drosophila melanogaster* to test the hypothesis that melanism and desiccation tolerance are functionally linked. Neither experiment yielded results consistent with our predictions, so we reject this simple hypothesis. Other physiological variables, such as body size and glycogen levels, can affect water balance, but these did not differ in pigmentation-selected populations. Disagreement between our findings and those of other labs may reflect differences in founding populations and details of experimental evolution procedures. Melanism has multiple potential functions in insects, which may be responsible for biogeographic clines in natural populations.

## Supporting Information

S1 Fig Carbohydrate content of pigmentation-selected populations after 52 generations of artificial selection. Upper panel, females; lower panel, males. Each bar represents mean (±SE) for a replicate population. Open bars, L_PIG_; gray bars, C_PIG_; black bars, D_PIG_. Data are means (±SE). For each sex, n = 8 per replicate.
(JPG)

S2 Fig Tergite area of pigmentation-selected populations after 40 generations of artificial selection. Upper panel, females; lower panel, males. Open bars, L_PIG_; gray bars, C_PIG_; black bars, D_PIG_. For each sex, n = 9–10 per replicate population.
(JPG)

S3 Fig Tergite area of desiccation-selected (D) populations and fed (F) controls after >140 generations of natural laboratory selection. Upper panel, females; lower panel, males. Open symbols, F flies; filled symbols, D flies. Each bar represents a different replicate population. For each sex, n = 15–20 per replicate population.
(JPG)

S4 Fig Water-loss rates of desiccation-selected (D) populations and fed (F) controls after ~140 generations of natural laboratory selection. Upper panel, females; lower panel, males. Open symbols, F flies; filled symbols, D flies. Each bar represents a different replicate population. For each sex, n = 6 groups of 10–20 flies each per replicate population.
(JPG)

S1 Table ANOVA results for grey scores of pigmentation-selected populations. For each sex, n = 9–10 per flies replicate population.
(DOCX)

S2 Table ANOVA results for grey-scale pigmentation scores for desiccation-selected and fed control populations. n = 15–20 flies per replicate population.
(DOCX)

S3 Table Results of pairwise comparisons (using log-ranks tests) of desiccation resistance in replicated desiccation-selected (D) and fed control (F) populations. Table entries are P-values. Significant differences (after sequential Bonferroni correction) are in bold font.
(DOCX)

S4 Table ANOVA results for wet mass, dry mass and water content of pigmentation-selected flies and controls. For each sex, n = 29–30 flies per replicate population.
(DOCX)

S5 Table Nested ANOVA results for carbohydrate content of pigmentation-selected populations and controls. For each sex, n = 6–8 flies per replicate population.
(DOCX)

S6 Table Nested ANOVA results for tergite area of pigmentation-selected populations and controls. For each sex, n = 9–10 flies per replicate population.
(DOCX)

S7 Table Nested ANOVA results for tergite area of desiccation-selected and fed control populations. For each sex, n = 9–10 flies per replicate population.
(DOCX)

S8 Table Nested ANOVA results for water-loss rate of desiccation-selected and fed control populations. (DOCX)

## References

[1] Mani MS. Ecology and Biogeography of High Altitude Insects Series Entomologia v.4. Junk N.V. Publishers, The Hague, Netherlands 1968.

[2] Majerus MEN. Melanism: Evolution in Action. Oxford University Press, Oxford, UK 1998.

[3] True JR. Insect melanism: The molecules matter. TREE 2003;18: 640–647.

[4] Gray SM, Mckinnon JS. Linking color polymorphism maintenance and speciation. TREE 2007;22: 71–79. 17055107 10.1016/j.tree.2006.10.005

[5] Kronforst MR, Barsh GS, Kopp A, Mallet J, Monteiro A, Mullen SP, et al Rosenblum EB, Schneider CJ, Hoekstra HE. Unraveling the thread of nature’s tapestry: the genetics of diversity and convergence in animal pigmentation. Pigment Cell Melanoma Res. 2012;25: 411–433. 10.1111/j.1755-148X.2012.01014.x 22578174

[6] Wittkopp PJ, Beldade P. Development and evolution of insect pigmentation: Genetics mechanisms and the potential consequences of pleiotropy. Semin. Dev. Biol. 2009;20: 65–71. 10.1016/j.semcdb.2008.10.002 18977308

[7] Kalra B, Parkash R, Aggarwal DD. Divergent mechanism for water conservation in *Drosophila* species. Entomol Exper Appl. 2014;151: 43–56.

[8] Dombeck I, Jaenike J. Ecological genetics of abdominal pigmentation in *Drosophila falleni*: A pleiotropic link to nematode parasitism. Evolution 58:587–596. 2004 15119442

[9] Rajpurohit S, Parkash R, Ramniwas S. Body melanization and its adaptive role in thermoregulation and tolerance against desiccating conditions in drosophilids. Entomol Res. 2008a;38: 49–60.

[10] Johnson RA, Kaiser A, Quinlan MC, Sharp W. Effects of cuticular abrasion and recovery on water loss rates in queens of the desert harvester ant *Messor pergandei*. J Exp Biol. 2011;214: 3495–3506. 10.1242/jeb.054304 21957113

[11] Bastide H, Yassin A, Johanning EJ, Pool JE. Pigmentation in *Drosophila melanogaster* reaches its maximum in Ethiopia and correlates most strongly with ultra-violet radiation in sub-Saharan Africa. BMC Evol Biol. 2014;14: 179 10.1186/s12862-014-0179-y 25115161 PMC4236528

[12] Kalmus H. The resistance to desiccation of *Drosophila* mutants affecting body color. Proc R Soc B. 1941;130: 185–201.

[13] Brisson JA, Toni DCD, Duncan I, Templeton AR. Abdominal pigmentation variation in *Drosophila polymorpha*: Geographic variation in the trait and underlying phylogeography. Evolution 2005;59: 1046–1059. 16136804

[14] Rajpurohit S, Parkash R, Ramniwas S, Singh S. Variations in body melanization, ovariole number and fecundity in highland and lowland populations of *Drosophila melanogaster* from the Indian subcontinent. Insect Sci. 2008b;15: 553–561.

[15] Rajpurohit S, Nedved N. Clinal variation in fitness related traits in tropical drosophilids of the Indian subcontinent. J Therm Biol. 2013;38: 345–354.

[16] Parkash R, Rajpurohit S, Ramniwas S. Impact of darker, intermediate and lighter phenotypes of body melanization on desiccation resistance in *Drosophila melanogaster*. J Insect Sci. 2009; 9:49. 10.1673/031.009.4901 PMC3011941 20050769

[17] Rajpurohit S, Nedved O, Gibbs AG. Meta-analysis of geographical clines in desiccation tolerance of Indian drosophilids. Comp Biochem Physiol A Mol Integr Physiol. 2013;164: 391–398. 10.1016/j.cbpa.2012.11.013 23182926

[18] Parkash R, Rajpurohit S, Ramniwas S. Changes in body melanisation and desiccation resistance in highland vs. lowland populations of *D*. *melanogaster*. J Insect Physiol. 2008b;54: 1050–1056. 18519137 10.1016/j.jinsphys.2008.04.008

[19] Parkash R. Testing the melanism-desiccation hypothesis: A case study in Darwinian evolution Pp. 279–306 in Nature at Work: Ongoing Sage of Evolution. Sharma V.P., ed. The National Academy of Sciences, India 2010.

[20] Ramniwas S, Kajla B, Dev K, Parkash R. Direct and correlated responses to laboratory selection for body melanization in *Drosophila melanogaster*: support for the melanization-desiccation resistance hypothesis. J Exp Biol. 2013;216: 1244–1254. 10.1242/jeb.076166 23239892

[21] Gibbs AG, Rajpurohit S. Water-proofing properties of cuticular lipids Pp. 100–120 in Blomquist G.J. and Bagneres A.G., eds. Insect Lipids; Biology, Biochemistry and Chemical Biology (eds). Cambridge Publisher, Cambridge, UK 2010.

[22] Wittkopp PJ, Smith-Winberry G, Arnold LL, Thompson EM, Cooley AM, Yuan DC, et al Local adaptation for body color in *Drosophila americana*. Heredity 2011;106: 592–602. 10.1038/hdy.2010.90 20606690 PMC3183901

[23] Matute DR, Harris A. The influence of abdominal pigmentation on desiccation and ultraviolet resistance in two species of *Drosophila*. Evolution 2013; 67: 2451–2460. 10.1111/evo.12122 23888866

[24] Parkash R, Kalra B, Sharma V. Changes in cuticular lipids, water loss and desiccation resistance in a tropical drosophilid—Analysis of within population variation. Fly 2008a;2: 189–197. 18719406 10.4161/fly.6619

[25] Rose MR, Nusbaum TJ, Chippindale AK. Laboratory evolution: The experimental wonderland and the Cheshire cat syndrome Pp. 221–241 in Rose M.R. and Lauder G.V., eds. Adaptation. Academic Press, San Diego, USA 1996.

[26] Gibbs AG. Laboratory selection for the comparative physiologist. J Exp Biol. 1999;202: 2709–2718. 10504307 10.1242/jeb.202.20.2709

[27] Folk DG, Bradley TJ. Adaptive evolution in the lab: unique phenotypes in fruit flies comprise a fertile field of study. Integr Comp Biol. 2005;45: 492–499. 10.1093/icb/45.3.492 21676794

[28] Burke MK, Rose MR. Experimental evolution with *Drosophila*. Am J Physiol Regul Integr Comp Physiol. 2009;296: R1847–R1854. 10.1152/ajpregu.90551.2008 19339679

[29] Dykhuizen DE, Dean AM. Experimental evolution from the bottom up Pp. 67–88 in Garland T. and Rose M.R., eds. Experimental Evolution. University of California Press, Berkeley, USA 2009.

[30] Gibbs AG, Gefen E. Physiological adaptation and laboratory selection In Garland T. and Rose M.R., eds. Experimental Evolution. Pp. 523–530. University of California Press, Berkeley, USA 2009.

[31] Lynch CB. Clinal variation in cold adaptation in *Mus domesticus*: verification of predictions from laboratory populations. Am Nat. 1992;139: 1219–1236.

[32] Rajpurohit S, Gibbs AG. Selection of body tergite pigmentation and correlated responses in trident: a case study in *Drosophila melanogaster*. Biol J Linn Soc Lond. 2012;106: 287–294.

[33] Rose MR, Vu LN, Park SU, Graves JL. Selection on stress resistance increases longevity in *Drosophila melanogaster*. Exper Gerontol. 1992;27: 241–250. 1521597 10.1016/0531-5565(92)90048-5

[34] Archer MA, Bradley TJ, Mueller LD, Rose MR. Using experimental evolution to study the physiological mechanisms of desiccation resistance in *Drosophila melanogaster*. Physiol. Biochem. Zool. 2007;80: 386–398. 17508334 10.1086/518354

[35] Gefen E, Marlon AJ, Gibbs AG. Selection for desiccation resistance in adult *Drosophila melanogaster* affects larval development and metabolite accumulation. J Exp Biol. 2006;209: 3293–3300. 16916965 10.1242/jeb.02397

[36] David JR, Capy P, Gauthler JP. Abdominal pigmentation and growth temperature in Drosophila melanogaster. Similarities and differences in the norms of reactions of successive segements. J Evol Biol. 1990;3: 429–445.

[37] Rajpurohit S, Marlon AJ. Pigmentation scoring method for *Drosophila*. Drosoph Inf Serv. 2011;94: 134.

[38] Gibbs AG, Chippindale AK, Rose MR. Physiological mechanisms of evolved desiccation resistance in *Drosophila melanogaster*. J Exp Biol. 1997;200: 1821–1832. 9225453 10.1242/jeb.200.12.1821

[39] Slocumb ME, Regalado JM, Yoshizawa M, Neely GG, Masek P, Gibbs AG, et al Enhanced sleep is an evolutionarily adaptive response to starvation stress in *Drosophila*. PLoS One 2015;10: e0131275 10.1371/journal.pone.0131275 26147198 PMC4493134

[40] Folk DG, Han C, Bradley TJ. Water acquisition and partitioning in *Drosophila melanogaster*: effects of selection for desiccation resistance. J Exp Biol. 2001;204: 3323–3331. 11606606 10.1242/jeb.204.19.3323

[41] Watt WB. Adaptive significance of pigment polymorphism in *Colias butterflies*. II. Thermoregulation and photoperiodically controlled melanin variation in *Colias eurytheme*. Proc Natl Acad Sci U S A 1969;63: 767–774. 16591777 10.1073/pnas.63.3.767 PMC223518

[42] Brakefield PM, Willmer PG. The basis of thermal melanism in the ladybird *Adalia bipunctata*: differences in reflectance and thermal properties between morphs. Heredity 1985;54: 9–14.

[43] Willmer PG, Unwin DW. Field analyses of insect heat budgets—reflectance, size and heating rates. Oecologia 1981;50: 250–255. 28311097 10.1007/BF00348047

[44] Prange HD. Evaporative cooling in insects. J Insect Physiol. 1996;42: 493–499.

[45] Chown SL, Gibbs AG, Hetz SK, Klok CJ, Lighton JRB, Marais E. Discontinuous gas exchange in insects, a clarification of hypothesis and approaches. Physiol Biochem Zool. 2006;79: 333–343. 16555192 10.1086/499992

[46] Quinlan MC, Gibbs AG. Discontinuous gas exchange in terrestrial insects. Respir Physiol Neurobiol. 2006;154: 18–29. 16870512 10.1016/j.resp.2006.04.004

[47] Williams AE, Bradley TJ. The effect of respiratory pattern on water loss in desiccation-resistant *Drosophila melanogaster*. J Exp Biol. 1998;201: 2953–2959. 9866880 10.1242/jeb.201.21.2953

[48] Marron MT, Markow TA, Kainand KJ, Gibbs AG. Effects of starvation and desiccation on energy metabolism in desert and mesic *Drosophila*. J Insect Physiol. 2003;49: 261–270. 12770001 10.1016/s0022-1910(02)00287-1

[49] Parkash R, Aggarwal DD, Kalra B. Co-adapted changes in energy metabolites and body color phenotypes for resistance to starvation and desiccation in latitudinal populations of *D*. *melanogaster*. Evol Ecol. 2012;26: 149–169.

[50] Telonis-Scott M, Guthridge KM, Hoffmann AA. A new set of laboratory-selected Drosophila melanogaster lines for the analysis of desiccation resistance: response to selection, physiology and correlated responses. J Exp Biol. 2006;209: 1837–1847. 16651550 10.1242/jeb.02201

[51] Shakhmantsir I, Massad NL, Kennell JA. Regulation of cuticle pigmentation in *Drosophila* by the nutrient sensing insulin and TOR signaling pathways. Dev Dyn. 2014; 243: 393–401. 10.1002/dvdy.24080 24133012

[52] Chippindale AK, Gibbs AG, Sheik M, Yee KJ, Djawdan M, Bradley TJ, et al Resource acquisition and the evolution of stress resistance in *Drosophila melanogaster*. Evolution 1998;52: 1342–1352. 28565385 10.1111/j.1558-5646.1998.tb02016.x

[53] Williams AE, Rose MR, Bradley TJ. CO2 release patterns in Drosophila melanogaster: the effect of selection for desiccation resistance. J. exp. Biol. 1997;200: 615–624. 9057311 10.1242/jeb.200.3.615

[54] Foley BR, Telonis-Scott M. Quantitative genetics analysis suggests casual association between cuticular hydrocarbon composition and desiccation survival in *Drosophila melanogaster*. Heredity 2011;106: 68–77. 10.1038/hdy.2010.40 20389309 PMC2905492

[55] Gibbs AG, Louie AK, Ayala JA. Effects of temperature on cuticular lipids and water balance in a desert *Drosophila*: Is thermal acclimation beneficial? J Exp Biol. 1998;201: 71–80. 9390938 10.1242/jeb.201.1.71

[56] Gibbs AG, Matzkin LM. Evolution of water balance in the genus *Drosophila*. J Exp Biol. 2001;204: 2331–2338. 11507115 10.1242/jeb.204.13.2331

[57] Chung H, Loehlin DW, Dufour HD, Vacarro K, Millar JG, Carroll SB. A single gene affects both ecological divergence and mate choice in *Drosophila*. Science 2014; 343: 1148–1151. 10.1126/science.1249998 24526311

[58] Parkash R, Sharma V, Kalra B. Climatic adaptations of body melanisation in *Drosophila melanogaster* from western Himalayas. Fly 2008c;2: 111–117. 18820467 10.4161/fly.6351

[59] David JR, Capy P. Genetic variation of *Drosophila melanogaster* natural populations. Trends in Genetics 1988;4: 106–111. 3149056 10.1016/0168-9525(88)90098-4

[60] Caracristi G, Schlotterer C. Genetic differentiation between American and European Drosophila melanogaster populations could be attributed to admixture of African alleles. Mol Biol Evol 2003;20: 792–799. 12679536 10.1093/molbev/msg091

[61] Campo D, Lehmann K, Fieldsted C, Souaiaia T, Kao J, Nuzhdin SV. Whole genome sequencing of two North American *Drosophila melanogaster* populations reveals genetic differentiation and positive selection. Mol Ecol. 2013;22: 5084–5097. 10.1111/mec.12468 24102956 PMC3800041

[62] Keller A. *Drosophila melanogaster*’s history as a human commensal. Curr Biol. 2007;17: 77–R81. 10.1016/j.cub.2006.12.031 17276902

[63] Laurent SJY, Werzner A, Excoffier L, Stephan W. Approximate *Bayesian* analysis of *Drosophila melanogaster* polymorphism data reveals a recent colonization of Southeast Asia. Mol Biol Evol. 2011;28: 2041–2051. 10.1093/molbev/msr031 21300986

[64] Begun DJ, Aquadro CF. African and North American populations of *Drosophila melanogaster* are very different at the DNA level. Nature 1993;365: 540–550. 10.1038/365548a0 8413609

[65] Dobzhansky T. 1937 Genetics and the origin of species. New York, NY: Columbia University Press.

[66] Krimbas CB, Powell JR. Drosophila Inversion Polymorphism. Boca Raton, FL: CRC Press 1992.

[67] Hoffmann AA, Sgro CM, Weeks AR. Chromosomal inversion polymorphisms and adaptation. TREE 2004;19: 482–488. 16701311 10.1016/j.tree.2004.06.013

[68] Ayala D, Ullastres A, Gonzalez J. Adaptation through chromosomal inversions in Anopheles. Front Genet. 2014;5: 129 10.3389/fgene.2014.00129 24904633 PMC4033225

[69] Langley CH, Lazzaro BO, Phillips W, Heikkinen E, Braverman JM. Linkage disequilibria and the site frequency spectra in the su(s) and su(w(a) regions of the *Drosophila melanogaster* X chromosome. Genetics 2000;156: 1837–1852. 11102378 10.1093/genetics/156.4.1837 PMC1461393

