# Supplementary figures and images for: An Experimental Evolution Test of the Relationship between Melanism and Desiccation Survival in Insects
Source: PLoS One. 2016 Sep 22;11(9):e0163414. doi: 10.1371/journal.pone.0163414 (PMC5033579; doi:10.1371/journal.pone.0163414)

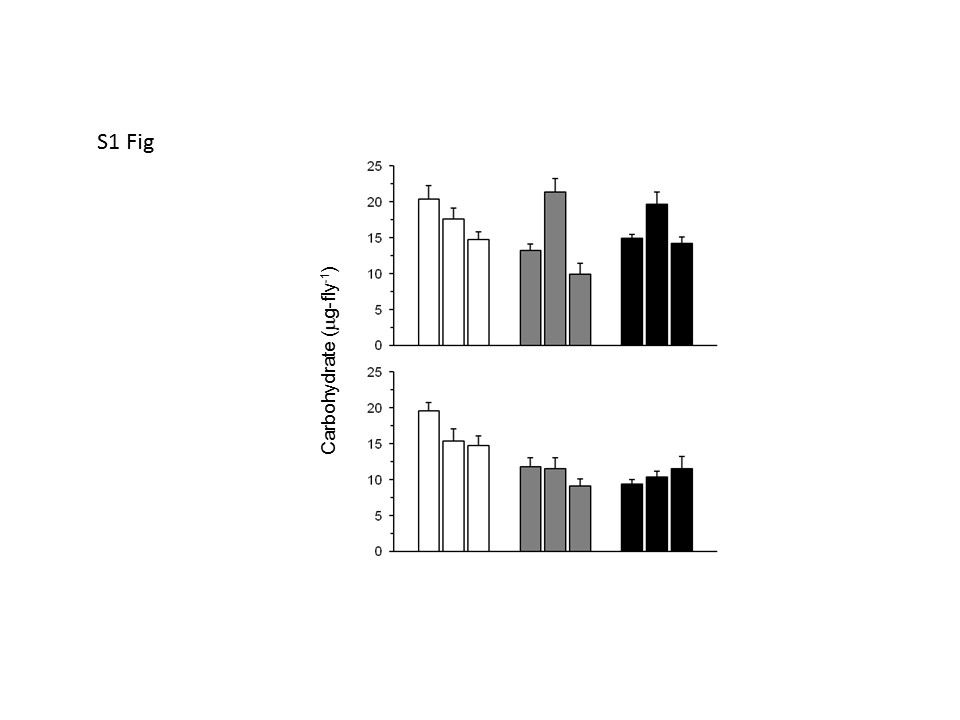

Supplement: S1 Fig — Upper panel, females; lower panel, males. Each bar represents mean (±SE) for a replicate population. Open bars, LPIG; gray bars, CPIG; black bars, DPIG. Data are means (±SE). For each sex, n = 8 per replicate. (JPG) [file pone.0163414.s001.jpg]

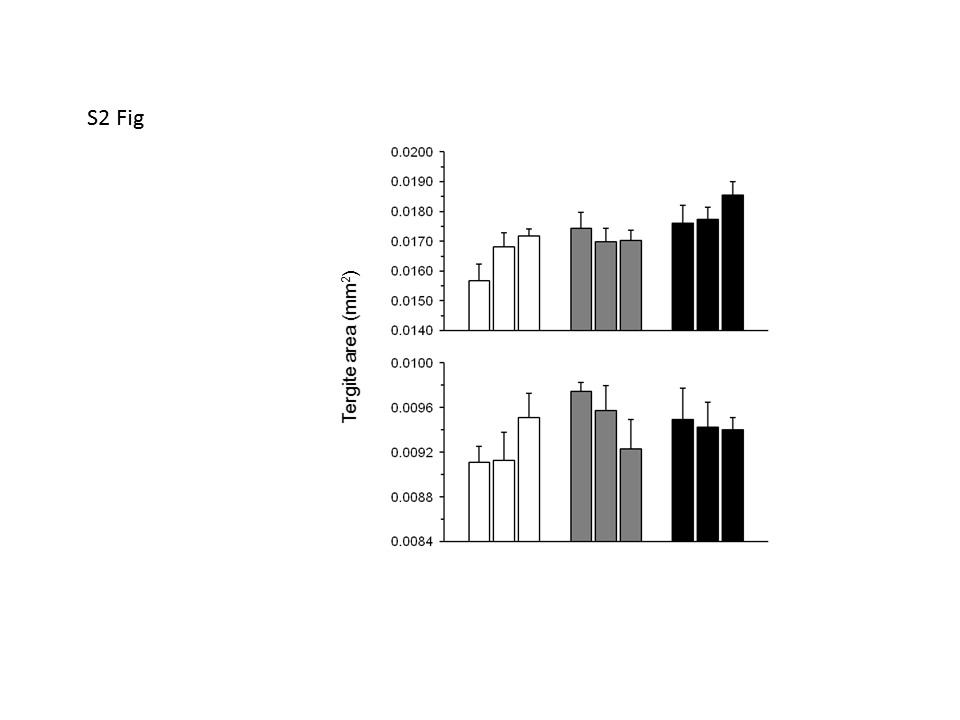

Supplement: S2 Fig — Upper panel, females; lower panel, males. Open bars, LPIG; gray bars, CPIG; black bars, DPIG. For each sex, n = 9–10 per replicate population. (JPG) [file pone.0163414.s002.jpg]

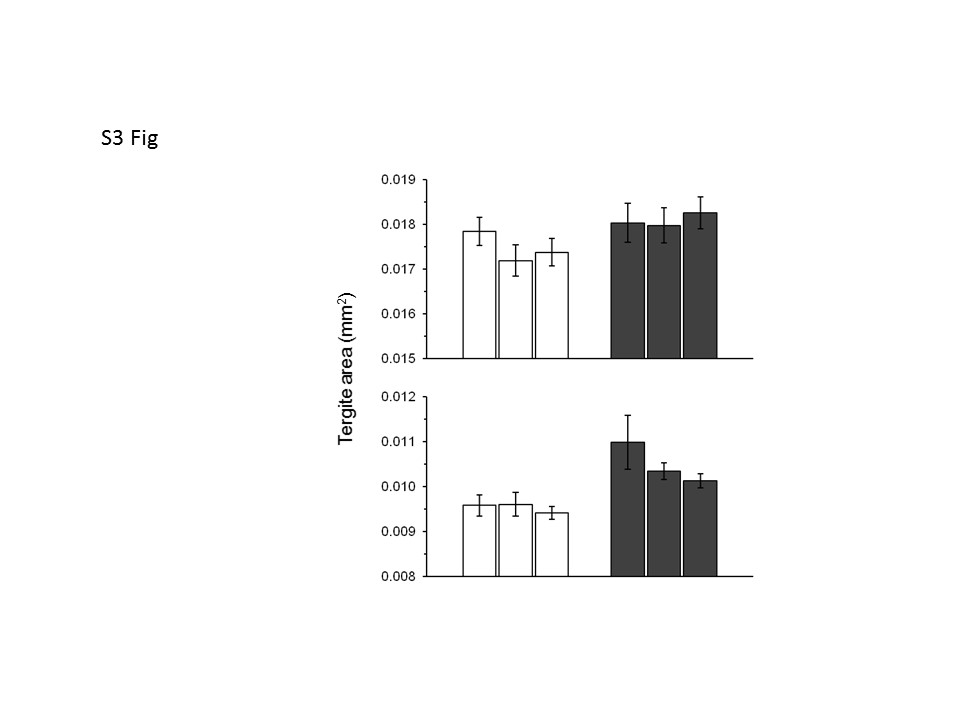

Supplement: S3 Fig — Upper panel, females; lower panel, males. Open symbols, F flies; filled symbols, D flies. Each bar represents a different replicate population. For each sex, n = 15–20 per replicate population. (JPG) [file pone.0163414.s003.jpg]

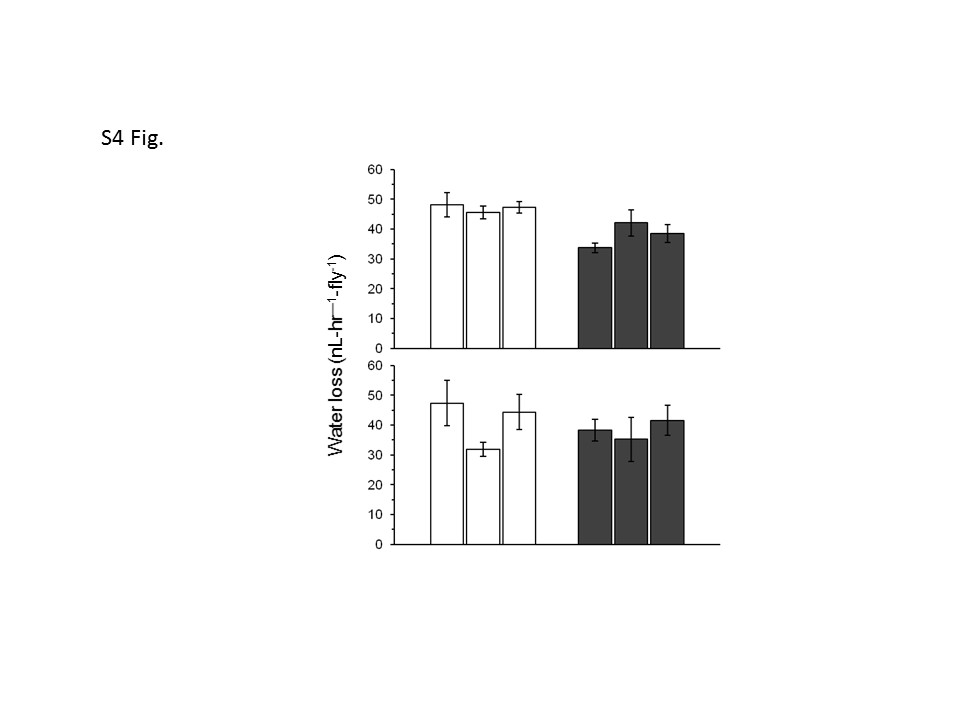

Supplement: S4 Fig — Upper panel, females; lower panel, males. Open symbols, F flies; filled symbols, D flies. Each bar represents a different replicate population. For each sex, n = 6 groups of 10–20 flies each per replicate population. (JPG) [file pone.0163414.s004.jpg]
