# Supplementary material for: An Experimental Evolution Test of the Relationship between Melanism and Desiccation Survival in Insects
Source: PLoS One. 2016 Sep 22;11(9):e0163414. doi: 10.1371/journal.pone.0163414 (PMC5033579; doi:10.1371/journal.pone.0163414)
Supplement: S1 Table — For each sex, n = 9–10 per flies replicate population. (DOCX) [file pone.0163414.s005.docx]

**Table S1.** ANOVA results for grey scores of pigmentation-selected populations. For each sex, n= 9-10 per flies replicate population.

| Parameter | Effect (F/R) | | SS | DF | MS | F | p |
| --- | --- | --- | --- | --- | --- | --- | --- |
| selection | Fixed | 15628 | | 2 | 7814 | 99.91 | **0.000025** |
| replicate(selection) | Random | 469 | | 6 | 78 | 0.67 | 0.68 |
| sex | Fixed | 6783 | | 1 | 6783 | 58.13 | **0.0003** |
| replicate(selection*sex) | Random | 700 | | 6 | 117 | 3.24 | **0.005** |
| selection*sex | Fixed | 578 | | 2 | 289 | 2.47 | 0.16 |
| Error |  | 5798 | | 161 | 36 |  |  |
