# Supplementary material for: An Experimental Evolution Test of the Relationship between Melanism and Desiccation Survival in Insects
Source: PLoS One. 2016 Sep 22;11(9):e0163414. doi: 10.1371/journal.pone.0163414 (PMC5033579; doi:10.1371/journal.pone.0163414)
Supplement: S2 Table — n = 15–20 flies per replicate population. (DOCX) [file pone.0163414.s006.docx]

**Table S2.** ANOVA results for grey-scale pigmentation scores for desiccation-selected and fed control populations. n = 15-20 flies per replicate population.

| Parameter | Effect (F/R) | SS | df | MS | F | | p | |  |
| --- | --- | --- | --- | --- | --- | --- | --- | --- | --- |
| Selection | Fixed | 17 | 1 | 17 | | 1.0 | | 0.36 | |
| Replicate(Selection) | Random | 64 | 4 | 16 | | 1.4 | | 0.37 | |
| Sex | Fixed | 4067 | 1 | 4067 | | 363.9 | | **0.00005** | |
| Replicate(Selection*Sex) | Random | 45 | 4 | 11 | | 0.5 | | 0.75 | |
| Selection*Sex | Fixed | 37 | 1 | 37 | | 3.3 | | 0.14 | |
| Error |  | 4636 | 198 | 23 | |  | |  | |
