# Supplementary material for: An Experimental Evolution Test of the Relationship between Melanism and Desiccation Survival in Insects
Source: PLoS One. 2016 Sep 22;11(9):e0163414. doi: 10.1371/journal.pone.0163414 (PMC5033579; doi:10.1371/journal.pone.0163414)
Supplement: S3 Table — Table entries are P-values. Significant differences (after sequential Bonferroni correction) are in bold font. (DOCX) [file pone.0163414.s007.docx]

**Table S3.** Results of pairwise comparisons (using log-ranks tests) of desiccation resistance in replicated desiccation-selected (D) and fed control (F) populations. Table entries are P-values. Significant differences (after sequential Bonferroni correction) are in bold font.

|  | FA | FB | FC | DA | DB | DC |
| --- | --- | --- | --- | --- | --- | --- |
| FA | --- |  |  |  |  |  |
| FB | 0.80008 | --- |  |  |  |  |
| FC | 0.48727 | 0.47885 | --- |  |  |  |
| DA | **0.00375** | **0.00006** | **0.00074** | --- |  |  |
| DB | **0.00192** | **0.00002** | **0.00031** | 0.17789 | --- |  |
| DC | **0.00389** | **0.00007** | **0.00088** | 0.04609 | 0.0099 | --- |
