# Supplementary material for: An Experimental Evolution Test of the Relationship between Melanism and Desiccation Survival in Insects
Source: PLoS One. 2016 Sep 22;11(9):e0163414. doi: 10.1371/journal.pone.0163414 (PMC5033579; doi:10.1371/journal.pone.0163414)
Supplement: S4 Table — For each sex, n = 29–30 flies per replicate population. (DOCX) [file pone.0163414.s008.docx]

| **Table S4**. ANOVA results for wet mass, dry mass and water content of pigmentation-selected flies and controls. For each sex, n = 29-30 flies per replicate population. | | | | | | |
| --- | --- | --- | --- | --- | --- | --- |
|  |  |  |  |  |  |  |
|  |  |  |  |  |  |  |
| A. Wet mass |  |  |  |  |  |  |
|  | Effect (F/R) | SS | df | MS | F | p |
| Selection | Fixed | 0.7991 | 2 | 0.3996 | 1.759 | 0.25 |
| Sex | Fixed | 10.0277 | 1 | 10.0277 | 489.548 | **0.000001** |
| Selection*Sex | Fixed | 0.0508 | 2 | 0.0254 | 1.240 | 0.35 |
| Replicate(Selection) | Random | 1.3628 | 6 | 0.2271 | 11.089 | **0.005** |
| Replicate*Sex(Selection) | Random | 0.1229 | 6 | 0.0205 | 2.669 | **0.015** |
| Error |  | 3.9914 | 520 | 0.0077 |  |  |
|  |  |  |  |  |  |  |
|  |  |  |  |  |  |  |
| B. Dry mass |  |  |  |  |  |  |
|  | Effect (F/R) | SS | df | MS | F | p |
| Selection | Fixed | 0.26523 | 2 | 0.13262 | 7.187 | **0.026** |
| Sex | Fixed | 0.79737 | 1 | 0.79737 | 1362.201 | **0.000000** |
| Selection*Sex | Fixed | 0.00766 | 2 | 0.00383 | 6.541 | **0.031** |
| Replicate(Selection) | Random | 0.11071 | 6 | 0.01845 | 31.522 | **0.00028** |
| Replicate*Sex(Selection) | Random | 0.00351 | 6 | 0.00059 | 0.872 | 0.52 |
| Error |  | 0.34897 | 520 | 0.00067 |  |  |
|  |  |  |  |  |  |  |
|  |  |  |  |  |  |  |
| C. Water content |  |  |  |  |  |  |
|  | Effect (F/R) | SS | df | MS | F | p |
| Selection | Fixed | 0.2443 | 2 | 0.1221 | 0.951 | 0.44 |
| Sex | Fixed | 5.1697 | 1 | 5.1697 | 326.272 | **0.000002** |
| Selection*Sex | Fixed | 0.0283 | 2 | 0.0142 | 0.894 | 0.46 |
| Replicate(Selection) | Random | 0.7704 | 6 | 0.1284 | 8.103 | **0.011** |
| Replicate*Sex(Selection) | Random | 0.0951 | 6 | 0.0158 | 3.284 | **0.0035** |
| Error |  | 2.5092 | 520 | 0.0048 |  |  |
