# Supplementary material for: An Experimental Evolution Test of the Relationship between Melanism and Desiccation Survival in Insects
Source: PLoS One. 2016 Sep 22;11(9):e0163414. doi: 10.1371/journal.pone.0163414 (PMC5033579; doi:10.1371/journal.pone.0163414)
Supplement: S5 Table — For each sex, n = 6–8 flies per replicate population. (DOCX) [file pone.0163414.s009.docx]

**Table S5**: Nested ANOVA results for carbohydrate content of pigmentation-selected populations and controls. For each sex, n = 6-8 flies per replicate population.

| Parameters | Effect (F/R) | SS | df | MS | F | p |
| --- | --- | --- | --- | --- | --- | --- |
| sel | Fixed | 491.18 | 2 | 245.59 | 2.23 | 0.19 |
| rep(sel) | Random | 662.28 | 6 | 110.38 | 2.22 | 0.18 |
| sex | Fixed | 457.45 | 1 | 457.45 | 9.20 | **0.023** |
| rep(sel*sex) | Random | 298.59 | 6 | 49.77 | 3.52 | **0.003** |
| sel*sex | Fixed | 135.36 | 2 | 67.68 | 1.36 | 0.33 |
| Error |  | 1753.89 | 124 | 14.14 |  |  |
