# Supplementary material for: An Experimental Evolution Test of the Relationship between Melanism and Desiccation Survival in Insects
Source: PLoS One. 2016 Sep 22;11(9):e0163414. doi: 10.1371/journal.pone.0163414 (PMC5033579; doi:10.1371/journal.pone.0163414)
Supplement: S6 Table — For each sex, n = 9–10 flies per replicate population. (DOCX) [file pone.0163414.s010.docx]

**Table S6.** Nested ANOVA results for tergite area of pigmentation-selected populations and controls. For each sex, n = 9-10 flies per replicate population.

| Parameter | Effect (F/R) | SS | df | MS | F | p |
| --- | --- | --- | --- | --- | --- | --- |
| selection | Fixed | 0.000019 | 2 | 0.000009 | 4.24 | 0.071 |
| replicate(selection) | Random | 0.000013 | 6 | 0.000002 | 1.81 | 0.24 |
| sex | Fixed | 0.002736 | 1 | 0.002736 | 2220.01 | **< 10^-6^** |
| replicate(selection*sex) | Random | 0.000007 | 6 | 0.000001 | 0.97 | 0.45 |
| selection*sex | Fixed | 0.000012 | 2 | 0.000006 | 4.80 | 0.057 |
| Error |  | 0.000205 | 161 | 0.000001 |  |  |
