# Supplementary material for: An Experimental Evolution Test of the Relationship between Melanism and Desiccation Survival in Insects
Source: PLoS One. 2016 Sep 22;11(9):e0163414. doi: 10.1371/journal.pone.0163414 (PMC5033579; doi:10.1371/journal.pone.0163414)
Supplement: S7 Table — For each sex, n = 9–10 flies per replicate population. (DOCX) [file pone.0163414.s011.docx]

**Table S7.** Nested ANOVA results for tergite area of desiccation-selected and fed control populations.

| Parameters | Effect (F/R) | SS | df | MS | F | p |
| --- | --- | --- | --- | --- | --- | --- |
| Selection | Fixed | 0.000032 | 1 | 0.000032 | 26.0 | **0.0069** |
| Replicate(Selection) | Random | 0.000005 | 4 | 0.000001 | 0.68 | 0.64 |
| Sex | Fixed | 0.003148 | 1 | 0.003148 | 1716 | **0.000002** |
| Replicate(Selection*Sex) | Random | 0.000007 | 4 | 0.000002 | 0.86 | 0.49 |
| Selection*Sex | Fixed | 0.000001 | 1 | 0.000001 | 0.80 | 0.42 |
| Error |  | 0.000422 | 198 | 0.000002 |  |  |
