# Supplementary material for: An Experimental Evolution Test of the Relationship between Melanism and Desiccation Survival in Insects
Source: PLoS One. 2016 Sep 22;11(9):e0163414. doi: 10.1371/journal.pone.0163414 (PMC5033579; doi:10.1371/journal.pone.0163414)
Supplement: S8 Table — (DOCX) [file pone.0163414.s012.docx]

**Table S8**: Nested ANOVA results for water-loss rate of desiccation-selected and fed control populations.

| **Parameters** | **SS** | ***d.f.*** | **MS** | **F** | ***p*** |
| --- | --- | --- | --- | --- | --- |
| Selection | 628.9 | 1 | 628.9 | 3.9422 | 0.12 |
| Replicate(Selection) | 638.2 | 4 | 159.5 | 1.2211 | 0.43 |
| Sex | 139.4 | 1 | 139.4 | 1.0673 | 0.36 |
| Replicate(Selection*Sex) | 522.6 | 4 | 130.7 | 1.0720 | 0.38 |
| Selection*Sex | 164.4 | 1 | 164.4 | 1.2584 | 0.32 |
| Error | 7312.6 | 60 | 121.9 |  |  |
